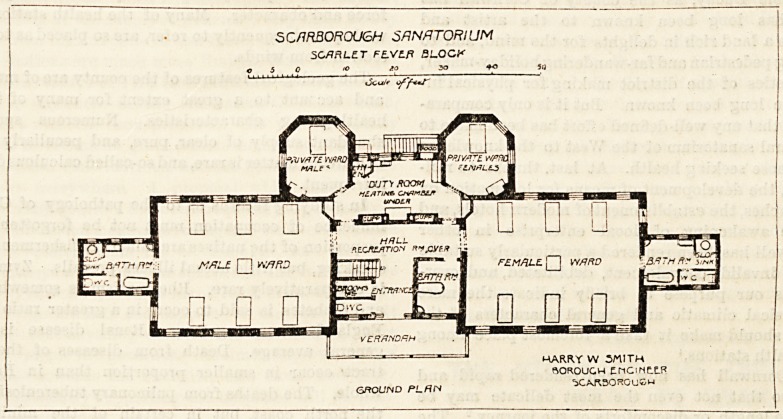# Scarborough Hospital for Infectious Diseases

**Published:** 1904-07-30

**Authors:** 


### HOSPITAL ADMINISTRATION.

### CONSTRUCTION AND ECONOMICS.

### SCARBOROUGH HOSPITAL FOR INFECTIOUS DISEASES.

THIS hospital stands on a site of seven acres, and is outside the town boundary to the north, being about two miles from the centre of the town. The site is 165 feet above the ordnance datum, and can be approached either from the Scalby or the Burniston Road. The land cost £1,200. The hospital consists of seven distinct blocks; these being the administrative; the lodge and discharge block (in one); the laundry; the scarlet fever wards; the enteric wards; the diphtheria wards, and the observation block. The laundry and the lodge are placed at opposite corners of the site and are near the east boundary; the administrative department lies towards the south-west corner, and the diphtheria wards are in a corresponding position near the north-west corner. The other blocks are centrally disposed. The scarlet fever whether for patients' or solely for nurses' use. A verandah is placed at the north-west front of the centre. To the south of the centre is the female ward for six beds and to the north is the male ward for the same number. Each ward has an oblong projection from the end which contains the bath-room, the closet, and the sink. The bath-room occupies the vestibule, so to speak, of this oblong, and, having a window on both sides, permits of cross-ventilation; but it would be infinitely preferable to have made an annexe of this part, and to have attached it to the ward by a short cross-ventilated passage as is the custom in almost every one of our modern hospitals. There are two private wards for one bed each, which, projecting eastwards, block out part of the ward, and hence one bed out of six has a one and the enteric one face east by south; and the observation block is placed behind the enteric one and at a sufficient distance from it. The various buildings seem well arranged with relation to each other, but we hardly understand why the scarlet fever and enteric blocks were not either made to face more to the south, or were not of a different plan, because, placed as they are, the male wards will not get much sunshine. The part of the lodge block containing the discharging-rooms is admirably arranged, as it enables the patients to undress in one room, and pass straight on to the bath-room, the discharging-room, and waiting-room.

The ground floor of the administrative department contains rooms for the medical officer of health, the dispensary, the matron's rooms, nurses' dining-room, and an excellent kitchen and offices. The first floor is given up to bedrooms for the resident staff of officers, nurses, and domestics, there being nine rooms for the former and six for the latter. The laundry department is also well arranged.

The scarlet fever block consists of a centre and two wings. The centre contains the entrance hall, corridor, nurses' duty room, a bath-room and a closet, both of the latter being incorporated with the centre. This plan cannot be pronounced quite satisfactory in the case of a bath-room, and it is objectionable in case of a closet, window on one side only. The private ward on the male side has a window on two aspects, hence cross-ventilation can be carried out. The other has windows on one side only. There are good inspection or observation windows looking into the six-bedded wards from the duty-room. The superficial space per bed would seem to be ample. There is a recreation-room over the central part of this block and this will prove a very useful adjunct.

The enteric block is smaller than the scarlet fever one, and it has the same drawbacks as regards the sanitary arrangements; and the same good points as regards space. In both of its private wards there is cross-ventilation. The observation ward has two one-bedded rooms and a duty room and there is a large well-lighted hall from which the closet and sink project. The diphtheria wards are in an old wood and iron building erected in 1892, and intended for small-pox patients. In all cases the elevations have a very nice appearance. They are of red brick with stone dressings. The roofs are slated. Apparently the windows are on the double-hung sash system with a large hopper over, and this is a decidedly good form for a hospital window, provided the top of the hopper is as close as possible to the ceiling. There are ventilators on the floor level, and exhaust ventilators in the ceiling, the latter acting on the "natural system." The floors of the wards are of polished oak blocks laid on concrete. These make a warm, firm, noiseless floor, and ought to be sanitary, *if* the joints can be kept quite close and free from dust. Terrazzo is used for the corridors and for the floors of the bath-rooms and lavatories. The sanitary fittings are of the latest and most approved kind. There is a steam disinfecting apparatus of the Washington Lyon type. The warming is by Shorland's grates, and these are supplemented where necessary by steam radiators on the low-pressure system.

The cost was about £14,000, and this sum included furnishing. There are 26 beds exclusive of those in the diphtheria wards.

The architect was Mr. Harry Smith, of Scarborough; the contractors for the brickwork were Messrs. Bastiman and Sons; Messrs. Bradford and Co. supplied the laundry machinery, and Messrs. Blakeborough and Rhodes fitted up the hot-water apparatus.

## Figures and Tables

**Figure f1:**